# Effectiveness of Different Doses of Tenoxicam in Preventing Propofol Injection Pain

**DOI:** 10.5152/TJAR.2021.1422

**Published:** 2022-02-01

**Authors:** Ökkeş Hakan Miniksar

**Affiliations:** Department of Anaesthesiology and Reanimation, University of Yozgat Bozok, Yozgat, Turkey

**Keywords:** Anaesthesia, injection pain, propofol, tenoxicam

## Abstract

**Objective:**

In this study, we aimed to investigate the effect of 2 different dosages of tenoxicam in the prevention of propofol injection pain.

**Methods:**

A total of 120 patients between the ages of 20-50 years who were scheduled for elective surgery were included in this prospective. Patients were randomly divided into 3 groups. Group 1 received 5 mL saline, group 2 received 10 mg tenoxicam in 5 mL saline, and group 3 received 20 mg tenoxicam in 5 mL saline intravenously as a pretreatment. Venous occlusion was applied for 60 seconds with a rubber tourniquet after the injection was completed. After injecting propofol, the pain at the injection site of the patient was questioned according to the Verbal Rating Scale.

**Results:**

The overall pain incidence during propofol injection was 85% in group 1, 75% in group 2, and 60% in group 3 (*P *= .039). While there was no significant difference between groups 1 and 2 (*P* = .264), there was a significant difference between groups 1 and 3 (*P* = .012). Moreover, there was a significant decrease in the level of severe pain in group 3 compared to group 1 (*P* = .008). There was no significant difference between the groups in terms of mild and moderate pain levels (*P* > .05).

**Conclusions:**

We found that 20 mg of tenoxicam pretreatment was effective in reducing the incidence and severity of propofol injection pain compared to the control saline group, but the 10 mg dose did not significantly reduce the injection pain.

**Main Points**

- Propofol injection pain is still an important problem in anaesthesia practice.
- In this study, the effect of tenoxicam pretreatment of pain during propofol injection was investigated.
- Pretreatment with 20 mg tenoxicam significantly reduced propofol injection compared to control saline group.
- The 10 mg dose did not significantly reduce the injection pain.

## Introduction

Propofol is an alkylphenol (2,6-diisopropylphenol) derivative and is the most widely used rapid onset intravenous (IV) anaesthetic agent for induction and maintenance of anaesthesia. Although propofol is an ideal IV anaesthetic agent, 28-90% of adults experience injection-related pain. Propofol injection pain (PIP) may not be a very serious complication but is remembered as an unpleasant experience before the operation. In a survey study, PIP was determined as the seventh most important problem in clinical anaesthesia practice.^1 – 3^ Factors affecting the incidence of PIP are injection site, injection speed, blood vessel size, propofol concentration in aqueous phase, buffering effect of blood, gender, and combined use of various drugs.^1,4,5^

Although the cause of PIP is not clearly revealed, some mechanisms have been suggested.^1^ Firstly, the propofol’s phenol groups can directly irritate the skin, mucous membranes, and venous wall intima and stimulate nociceptors and free nerve endings.^4^ Secondly, propofol can activate the kinin–kallikrein system by indirectly affecting the endothelium.^1,6, 7^ Studies based on these mechanisms have shown that nonsteroidal anti-inflammatory drugs (NSAIDs) can prevent PIP by reducing prostaglandin synthesis and inhibiting kinin cascade.^2,5-15^ In the literature, many NSAIDs have been used to reduce PIP.^10,11,16-20^ Among these drugs, acetaminophen and ketoroloc have been most frequently investigated in PIP.^5,10,11,16,17^ In a recent study, Başak et al.^12^ compared 2 mg, 4 mg, and 8 mg lornoxicam doses with the saline and reported that 4 mg lornoxicam was effective in reducing the incidence and severity of PIP. Identification of new methods and drugs in reducing injection pain and their incorporation into clinical practice could enhance PIP prevention and treatment practices.

Tenoxicam is an NSAID from the oxicam family that shows its rapid analgesic effect by non-selective cyclooxygenase (COX) inhibition.^6^ It has long been used effectively in many types of pain management.^6,21^ When the literature on PIP was reviewed, as far as we know, tenoxicam was investigated in injection pain in only 1 study.^6^ However, no comprehensive study showing the use of different doses of tenoxicam in PIP has been encountered. In this study, we aimed to investigate the effect of 2 different dosages of tenoxicam in prevention of PIP. 

## Methods

Written permission was obtained from the institution where the study was conducted and from the Local Clinical Research Ethics Committee (2018/21). The study was designed as prospective, randomized, double-blind, placebo-controlled study and was carried out in our hospital between June and September 2018 in accordance with the principles of the Helsinki Declaration. A total of 120 patients who underwent elective surgery under general anaesthesia with propofol induction and had the American Society of Anaesthesiologists (ASA) scores of 1-2 were included in the study. Patients that were under 20 or over 50 years old; had ASA scores of 3 and above; those with a history of allergic response to NSAID drugs or gastrointestinal bleeding; patients with bleeding diathesis, gastric and duodenal ulcer, or liver, kidney, heart, or pulmonary failure; those with chronic pain syndrome; diabetic patients; pregnant women; patients in lactation; emergency surgery; communication issues; unwillingness to participate in the study; and the ones that used analgesics within the last 24 hours were excluded from the study. 

The patients’ age, weight, medications, additional diseases, and drug allergy histories were evaluated and recorded during the preoperative visit. No premedication was applied and informed consent was obtained from all patients. Patients were taken to the operating room and monitored with 6-lead (I, II, III, aVL, aVR, and aVF) electrocardiogram, and their non-invasive blood pressure was monitored with pulse oximetry. Then nurses who did not participate in the study placed the 16-gauge IV cannula in a vein in the dorsum of the hand and saline infusion was initiated. Eight L dk-1 of oxygen/medical air was given through a face mask. It was explained that IV anaesthesia may cause pain during injection in patients would receive it. The patients were randomly divided into 3 groups: group 1 received 5 mL saline, group 2 received 10 mg tenoxicam group in 5 mL saline, and group 3 received 20 mg tenoxicam group in 5 mL saline as a pretreatment. The anaesthesiologist, who recorded the pain score during the study, was not aware of pretreatment medications. For all 3 groups, the volume of these drugs was brought up to an equal volume (5 mL) with 0.09% NaCl by an anaesthesiologist who did not know the groups’ distinctions. Venous occlusion was applied with a rubber tourniquet approximately 20 cm proximal to the vascular access for 30 seconds, and study drug (Oksamen L 20 mg flacon, MN İlaç, İstanbul, Turkey) was administered intravenously in 10 seconds.^6,12^ Venous occlusion was maintained for 60 seconds after completion of the injection.^5,17^ Then the tourniquet was relaxed and 25% of the 2.5 mg kg^−1^ propofol dose (Propofol-Lipuro 1% 10 mg mL^−1^, B Braun) was administered IV within 10 seconds. A different anaesthesiologist questioned patients about the presence of pain at the injection site based on the verbal pain scale (0 = absent, 1 = mild, 2 = moderate, and 3 = severe) and then the remaining dose of propofol was administered. Thirty seconds after the completion of the propofol injection, the full dose of rocuronium 0.6 mg kg^−1^ was administered IV in at least 10 seconds as a neuromuscular blocker. Surgical procedure was initiated after intubation. Electrocardiogram, systemic blood pressure, and SpO_2_ values of the patients were monitored throughout the operation. The injection site was checked for pain, edema, or allergic reaction during postoperative 24 hours.

Based on previous studies,^1,14^ the overall incidence of pain in patients receiving normal saline as placebo was considered to be about 75%, and a 35% reduction in incidence was considered clinically significant.^17^ With these assumptions, the required sample size at a 5% 2-sided significance level (G-power 3.1.9) with 85% power was calculated as 35 patients per group. Considering that some patients might be excluded from the study, it was decided to include 40 patients in each group.

## Statistical Analyses

Statistical analysis was performed using the Statistical Package for the Social Sciences (SPSS) software 20.0 program (IBM Corp.; Armonk, NY, USA). Normality analysis of variables was performed using the Kolmogorov–Smirnov test. The Mann–Whitney *U* test was used to evaluate age and weight variables that were not normally distributed. The categorical data were compared with each other by using Pearson’s chi-squared test and linear-by-linear association test (gender, ASA, and pain scores between groups). Quantitative data values were expressed in the tables as median (min-max). Categorical data were expressed as number (n) and percentage (%). Data were examined at a 95% CI and a value of *P* < .05 was considered statistically significant.

## Results

A total of 120 patients divided into 3 equal groups of 40 patients were included in the study. There was no significant difference between the groups in terms of demographic data (*P* > .05, Table 1).

To determine the effective dose of tenoxicam, 10 mg and 20 mg doses were compared with the control saline group. The distribution of groups according to their pain scores is shown in Table 2. Injection pain levels were compared in all 3 groups, and a statistically significant difference was found between them (*P* = .013). During propofol injection, the pain incidence was highest in group 1 (saline) (85%), followed by group 2 (tenoxicam 10 mg) (75%), and group 3 (tenoxicam 20 mg) (60%) (*P* = .039) (Table 2). When groups 1 and 2 were compared, no significant difference was found in terms of overall pain incidence (*P* = .264). However, there was a significant difference between groups 1 and 3 in terms of overall pain incidence (*P* = .012). There was a significant decrease in the level of severe pain in group 3 compared to group 1 (*P* = .008). Moreover, the number of patients without pain was significantly higher in group 3 compared to group 1 (*P* = .033). It was observed that as the dose of tenoxicam increased, the level of severe pain decreased and the number of patients without pain increased (linear-by-linear association: 15.3, *P* < .001). Severe pain was observed in 20 (15.87%) patients in total, while no pain was observed in 32 (25.39%) patients. There was no significant difference between the groups in terms of mild and moderate pain levels (*P* > .05). None of the patients had side effects related to the drug at the injection site during the injection and in the postoperative period.

## Discussion

In our study, we found that 20 mg tenoxicam pretreatment significantly reduced the overall pain incidence and severity of pain associated with IV propofol injection compared to the saline group, but the 10 mg dose of tenoxicam was not as effective in reducing PIP.

Although PIP is not a life-threatening problem associated with anaesthesia, it is an undesirable condition in clinical anaesthesia practice.^1^ Bronchospasms and myocardial ischemia may occur in patients as a result of stress and stimulation due to PIP during anaesthesia induction. In addition, the venous catheter may be dislodged due to limb movement.^22^ In our study, although severe PIP was observed in 20 patients, no injury was encountered.

The mechanism of PIP is still unclear. However, some mechanisms have been suggested.^1,14^ All phenols irritate the skin and mucous endothelium. The phenol groups of propofol also activate the kinin–kallikrein system, causing venous dilatation and hyperpermeability in that area, so bradykinin is released. Depending on this situation, a sensation of pain occurs.^1,4,7,15^

Many drugs from different groups as well as non-pharmacological methods such as dilution of propofol, increasing the injection speed and duration of the tourniquet, cooling or warming the drug, and using large-diameter vessels for injection have been investigated for their effects on preventing PIP.^1,4,14^ These pharmacological methods include: adding lidocaine to propofol; adjusting the pH of the propofol emulsion; using alfentanil, remifentanil, ketamine, metoclopramide, naphhamostat, granisetron, oral clonidine, cold saline solution, ketorolac, thiopental, magnesium sulfate, ephedrine, or nitroglycerin; using large blood vessels; and topical application of eutectic mixture of local anaesthetics or 60% lidocaine patch.^2,5-13^ In addition, among these drugs, many NSAIDs such as acetaminophen,^11,16^ ketorolac,^5,10,17^ lornoxicam,^12^ flurbiprofen,^18^ parecoxib,^19^ and diclofenac^20^ have been shown to prevent PIP by reducing prostaglandin synthesis and inhibiting kinin cascade. 

However, a comprehensive meta-analysis has suggested that due to heterogeneity of results and some NSAIDs causing PIP, they should not be recommended for prevention of PIP.^1^ Taş et al.^23^ compared preemptive use of 400 mg ibuprofen and 40 mg lidocaine and reported that ibuprofen was effective in preventing PIP.^23^ Borazan et al.^24^ reported that pretreatment with 2 mg kg-1 paracetamol was more effective than lidocaine in preventing PIP. The results of pretreatment with another NSAID, flurbiprofen axetil, are conflicting. While Nishiyama et al.^25^ have reported that flurbiprofen axetil was not effective in preventing PIP, another comprehensive meta-analysis^18^ reported that it was. Yağan et al.^6^ compared dexketoprofen and tenoxicam and similar to our study found that 20 mg tenoxicam pretreatment was effective in preventing PIP compared to the saline group.^6^ In the same study, it was stated that the overall pain incidence due to propofol injection was 76% in the saline group and 38% in the tenoxicam group.^6^ In our study, we found that the incidence of severe pain and general pain due to propofol was significantly lower with 20 mg of tenoxicam compared to the other groups, but the overall pain incidence was higher (60%). This difference in our findings may be due to sample size or tourniquet time applied for venous occlusion. In addition, the use of a rubber tourniquet is a practical procedure and can cause inconsistent pressures for different patients, a potential limitation of our study. In our study, after the pretreatment tenoxicam was given, tourniquet was applied for 60 seconds, whereas in Yağan et al’s study,^6^ they used a longer tourniquet time (2 minutes). Huang et al.^5^ compared the incidence of PIP at different tourniquet times (30, 60, and 120 seconds) with 10 mg ketorolac and found that the incidence of severe pain decreased as the tourniquet duration increased. In the same study, it was stated that applying a tourniquet for 60 seconds or more is effective in preventing PIP.^5^ Dexter et al.^10^ proposed that ketorolac, an NSAID, should remain in the vasculature for a long time in order to reduce prostaglandin synthesis and inhibit the kinin cascade. Similar to our study, Madan et al.^17^ reported that PIP was reduced by administering 10 mg ketorolac and applying a tourniquet for 60 seconds. Another study reported that combination of diclofenac and a shorter (30 seconds) tourniquet time was not an effective pretreatment to reduce PIP.^20^ Based on these results, we can interpret that the duration of tourniquet plays an important role in the pretreatment with NSIADs to prevent PIP.

Another important finding in our study was high incidence of pain. The overall pain incidence with other NSAIDs was 20% with 15 mg ketorolac,^5^ 24% with 1 mg/kg^−1^ paracetamol,^25^ and 20% with 8 mg lornoxicam.^12^ Another study compared 10 mg of ketorolac with 60 mg of lidocaine and reported overall incidence of pain as 28% and 24%, respectively.^17^ On the other hand, Taş et al.^23^ found that the overall pain incidence due to propofol injection with 400 mg ibuprofen pretreatment was 80%, which is higher than pain incidence reported in our study. Despite this high rate, the authors reported that the application of preemptive ibuprofen was effective in reducing PIP.^23^ Although in our study 20 mg of tenoxicam significantly reduced PIP compared to the saline group, its routine use in the prevention of PIP in anaesthesia practice is not considered ideal since it does not reduce the incidence of general pain due to propofol to a desired level. Multicenter, randomized studies with different agents are needed to evaluate the effectiveness of tenoxicam in PIP.

The review of the literature showed that pre-application of lidocaine before or concurrently with propofol injection using a tourniquet and injection through the antecubital vein are the most effective methods in preventing PIP.^1,14^ Saline and lidocaine have been used as control groups in the studies. In studies conducted with the saline group, the incidence of PIP was reported to be 100%,^23^ 96%,^17^ and 93.3%.^12^ In our study, similar to the literature, PIP developed in most of the patients from the saline group (85%). The incidence of PIP with lidocaine varies widely with some reporting 6%,^11^ while others reported rates up to 30%.^23^ In our study, the incidence of PIP seen with 20 mg tenoxicam was higher than in studies using lidocaine as a control group. Therefore, to further evaluate the effectiveness of tenoxicam in PIP, it should be compared with different agents with proven efficacy.

It has been determined that pretreatment with NSAIDs not only reduces PIP but also provides preemptive analgesia by reducing postoperative pain and postoperative opioid need.^9,23^ Various NSAIDs are widely used to provide preemptive analgesia.^9,21,26^ It has been stated that a 20 mg dose of tenoxicam can be safely used in the treatment of postoperative pain and in multimodal analgesia for preemptive purposes.^21,27^ In our study, the preemptive analgesia efficacy of tenoxicam on postoperative pain was not evaluated since the aim of the study was limited to evaluating injection pain. Future studies evaluating wider effects of tenoxicam as a preemptive analgesic may provide useful information. 

## Conclusion

We found that 20 mg of tenoxicam pretreatment was effective in reducing the incidence and severity of PIP compared to the control saline group, but 10 mg dose did not significantly reduce the injection pain. In addition, we observed that the incidence of general pain due to propofol was high with tenoxicam pretreatment. Future large-scale multicenter studies with longer tourniquet times and NSAID agents with known effectiveness as a control are needed to evaluate the effectiveness of tenoxicam in preventing PIP.

## Figures and Tables

**Table 1.** Demographic Characteristics of the Patients

	Group 1 (n = 40)	Group 2 (n = 40)	Group 3 (n = 40)	*P*
Saline n (%)	Tenoxicam, 10 mg n (%)	Tenoxicam, 20 mg n (%)
Age (years)^1^	37 (20-48)	38 (20-49)	40 (23-49)	.347^†^
Weight (kg)^1^	69 (56-96)	72 (56-98)	71 (55-98)	.721^†^
Sex (M/F)	18/22	22/18	18/22	.586^*^
ASA (I/II)	16/24	20/20	20/20	.585^*^

^*^Chi-squared test; ^†^Mann–Whitney *U* test.

^1^Presented by median (min-max).

**Table 2.** Overall Pain Incidence and Comparison of Groups According to Verbal Pain Scores During Propofol Injection

Saline n (%)	Tenoxicam, 10 mg n (%)	Tenoxicam, 20 mg n (%)
Verbal Pain Scale	Group 1 (n = 40)	Group 2 (n = 40)	Group 3 (n = 40)	*P*
0 (no pain)	6 (15%)	10 (25)	16 (40)^a^	.013^*^
1 (mild)	8 (20%)	10 (25%)	14 (35%)	
2 (moderate)	14 (35%)	14 (35%)	8 (20%)	
3 (severe)	12 (30%)	6 (15%)	2 (5%)^b^
Overall incidence	34 (85%)	30 (75%)^#^	24 (60%)^**^	.039^*^

Data are presented as number of patients (%), ^*^ *P* < .05 was considered significant, ^#^ *P* = .264 compared with group 1, ^**^ *P* = .012 compared with group 1.

^a^According to group 1, *P* = .033.

^b^According to group 1, *P* = .008.
